# The Cholera

**Published:** 1849-04

**Authors:** 


### THE CHOLERA.

The epidemic has continued to prevail in Great Britain throughout the winter. In New Orleans it appears at last to be vanishing. The *Delta* of the 1st of March says—

"According to the reports of the sextons to the board of health, the number of interments at the several city cemeteries, during the week ending on Saturday last, was seventy-four. Of this number there were but four of cholera. The number of deaths of that disease for the preceding week was over sixty. It is gratifying at last, to report, as we may now with confidence, the entire disappearance of this dreadful malady. For several weeks, since it ceased to be epidemic, the sporadic cases have furnished food for the worms, at the rate of from sixty to seventy deaths a week."

The pestilence has lingered long about Nashville without becoming epidemic, or spreading to the towns or country around. Dr. Buchanan, in a letter to us, dated 25th February, makes the following interesting statements respecting the disease.

"Up to the present time we have had about fifty deaths here from the cholera, and a much greater number of cases, the large proportion of which were mild at the commencement. Indeed, almost every case gave timely warning by some premonitory symptom, which, among the intelligent, was properly regarded and easily removed; but among the indigent and ignorant, often ran on to fatal collapse before assistance was sought. In our practice here we have found that opium and diffusible stimuli are *the* remedies to be relied on to avert the disease. Given in time, they are almost infallible; at least they have proved so under my own observation; and the success attending the bold administration of opium, even in almost hopeless cases, has been far beyond my expectation. But as I am now writing in too great haste to give satisfaction, I will conclude by saying that I will, if time will possibly permit, furnish you with a statement of the weather, the disease, and its treatment for your April number."

